# Late versus early response and depth of response are associated with improved outcomes in patients with newly diagnosed multiple myeloma enrolled in the TOURMALINE‐MM2 trial

**DOI:** 10.1002/jha2.759

**Published:** 2023-08-03

**Authors:** Paul G. Richardson, Thierry Facon, Christopher P. Venner, Nizar J. Bahlis, Fritz Offner, Darrell White, Lionel Karlin, Lotfi Benboubker, Eric Voog, Sung‐Soo Yoon, Kenshi Suzuki, Hirohiko Shibayama, Xiaoquan Zhang, Miguel Villarreal, Philip Twumasi‐Ankrah, Richard Labotka, Robert M. Rifkin, Sagar Lonial, Shaji K. Kumar, S. Vincent Rajkumar, Philippe Moreau

**Affiliations:** ^1^ Harvard Medical School Jerome Lipper Multiple Myeloma Center, Dana‐Farber Cancer Institute Boston Massachusetts USA; ^2^ Centre Hospitalier Universitaire (CHU) Lille Service des Maladies du Sang, University of Lille Lille France; ^3^ Cross Cancer Institute University of Alberta Edmonton Alberta Canada; ^4^ BC Cancer Vancouver Centre University of British Columbia Vancouver British Columbia Canada; ^5^ Arnie Charbonneau Cancer Institute University of Calgary Calgary Alberta Canada; ^6^ UZ Gent Gent Belgium; ^7^ QEII Health Sciences Center and Dalhousie University Halifax Nova Scotia Canada; ^8^ Hôpital Lyon Sud, Pierre‐Benite Lyon France; ^9^ CHRU TOURS Tours France; ^10^ Clinique Victor Hugo Le Mans France; ^11^ Department of Internal Medicine Seoul National University Hospital Seoul Republic of Korea; ^12^ Japan Red Cross Medical Center Shibuya‐ku Tokyo Japan; ^13^ Osaka University Graduate School of Medicine Suita Osaka Japan; ^14^ Takeda Development Center Americas, Inc. (TDCA) Lexington Massachusetts USA; ^15^ US Oncology Research – Rocky Mountain Cancer Centers Denver Colorado USA; ^16^ Department of Hematology and Medical Oncology Winship Cancer Institute Emory University School of Medicine Atlanta Georgia USA; ^17^ Mayo Clinic Rochester Minnesota USA; ^18^ Centre Hospitalier Universitaire de Nantes Nantes France

**Keywords:** depth of response, ixazomib, multiple myeloma, response kinetics

## Abstract

Deeper responses are associated with longer survival in multiple myeloma (MM); however, limited data exist on the impact of response kinetics on outcomes. We investigated progression‐free survival (PFS) and duration of response (DOR) by response depth and in early (best confirmed response 0–4 months; *n* = 424) versus late responders (best confirmed response >4 months; *n* = 281). Newly diagnosed patients enrolled in TOURMALINE‐MM2 receiving ixazomib‐lenalidomide‐dexamethasone (IRd) (*n* = 351) or placebo‐Rd (*n* = 354) were evaluated *post hoc*. Deeper responses were associated with longer PFS (complete response [CR] not reached [NR], very good partial response [VGPR] 37.2 months, partial response [PR] 16.4 months) and DOR (CR NR, VGPR 42.6 months, PR 15.4 months). Among patients with a PFS (*n* = 511) or DOR (*n* = 484) of ≥6 months who achieved ≥PR, median PFS was prolonged among late versus early responders receiving IRd (59.7 vs. 17.9 months) or placebo‐Rd (56.6 vs. 12.4 months), as was median DOR (IRd, NR vs. 20.9 months; placebo‐Rd, 58.2 vs. 11.7 months). While the treatment paradigm for newly diagnosed MM is treatment to progression, our findings suggest slowness of response to a proteasome inhibitor‐immunomodulatory drug‐steroid combination is not a negative predictor of outcome.

## Graphical abstract

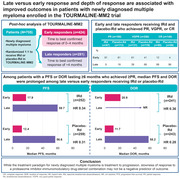

## 1 INTRODUCTION

The therapeutic landscape for multiple myeloma (MM) has improved with the introduction of novel agents, including proteasome inhibitors (PIs) and immunomodulatory drugs (IMiDs), which have led to an improvement in overall survival (OS) and higher response rates [1, 2, 3, 4]. There is now interest in quality and depth of response to treatment and how these parameters impact outcomes [5, 6].

A relationship between depth of response and clinical outcomes has been demonstrated consistently in MM [5, 7–9], with studies showing statistically superior outcomes, including prolonged progression‐free survival (PFS) [10, 11] and OS [12], in patients achieving at least a very good partial response (≥VGPR) during treatment. Conversely, the effects of response kinetics on outcomes in MM remain less well characterised [6, 12–16]. Nonetheless, the association between response kinetics and outcomes was evaluated in a post hoc analysis from the TOURMALINE‐MM1 study of ixazomib‐lenalidomide‐dexamethasone (IRd) versus placebo‐Rd in patients with relapsed/refractory MM (RRMM) [15]. In that analysis, Garderet et al. demonstrated longer PFS in patients who achieved ≥VGPR after 4 months (late responders) compared with those with ≥VGPR within 4 months (early responders) [15]. Similarly, a separate analysis from another study in patients with newly diagnosed MM (NDMM) receiving IMiD‐ or PI‐based induction showed that early responders who achieved a best objective response within 3 months had inferior PFS and OS compared with late responders with a response after 3 months [14].

Building on these observations, we have conducted an analysis to further investigate the relationship between depth and timing of response on clinical outcomes in MM. We have used PFS and duration of response (DOR) data from the randomised, double‐blind, phase 3 TOURMALINE‐MM2 trial (NCT01850524) of IRd versus placebo‐Rd in transplant‐ineligible patients with NDMM [17]. The primary results from TOURMALINE‐MM2 showed a clinically meaningful PFS benefit with IRd versus placebo‐Rd, although the difference between the two treatments was not statistically significant (median PFS, 35.3 vs. 21.8 months; hazard ratio [HR] 0.830; 95% confidence interval [CI]: 0.676–1.018; *p* = 0.073; median follow‐up, 53.3 and 55.8 months, respectively). Data from this post hoc analysis of the impact of response quality and kinetics on long‐term outcomes in TOURMALINE‐MM2 are presented.

## 2 METHODS

### 2.1 Patients

Full methods for TOURMALINE‐MM2 have been reported previously [17]. Adult patients with a new, confirmed diagnosis of symptomatic MM, according to International Myeloma Working Group (IMWG) 2011 criteria [18], and who were eligible for treatment with Rd but ineligible for autologous stem cell transplantation (ASCT), were enrolled. The trial was conducted in accordance with the International Conference on Harmonization Good Clinical Practice guideline and appropriate regulatory requirements. Local ethics committees or institutional review boards approved the protocol. All patients provided written informed consent.

### 2.2 TOURMALINE‐MM2 study design

Patients were randomised 1:1 to receive oral ixazomib 4 mg (*n* = 351) or matching placebo (*n* = 354) on days 1, 8, and 15 of 28‐day cycles. In addition, all patients received oral lenalidomide 25 mg on days 1–21 (10 mg for patients with creatinine clearance [CrCl] ≤60 or ≤50 mL/minute, depending on local prescribing information) and oral dexamethasone 40 mg (20 mg in patients aged > 75 years) on days 1, 8, 15, and 22. Randomisation was stratified by age, International Staging System (ISS) stage, and Brief Pain Inventory‐Short Form (BPI‐SF) worst pain score at screening. After 18 cycles, treatment was continued without dexamethasone and with reduced doses of ixazomib (3 mg) and lenalidomide (10 mg) until progressive disease (PD) or unacceptable toxicity (Figure S1) [17].

The primary endpoint was PFS, as assessed by an independent review committee (IRC). Prespecified key secondary endpoints were OS, complete response (CR) rate, and pain response rate. Other secondary endpoints included overall response rate (ORR), time to response, DOR, and time to progression. PFS in patients with expanded high‐risk cytogenetic abnormalities [del(17p), t(4;14), t(14;16), and/or amp(1q21)] was another secondary endpoint [17].

### 2.3 Assessments

In TOURMALINE‐MM2, progression and response assessments were based on central laboratory results and IMWG 2011 criteria [17, 18]. Response assessments were performed every cycle until PD, or every 4 weeks in patients who discontinued treatment prior to PD [17]. Cytogenetic abnormalities were assessed by a central laboratory using bone marrow aspirate samples taken at screening [17].

### 2.4 Statistical analysis

For this analysis, PFS (time from randomisation to first documentation of PD or death of any cause) and DOR (time from first documentation of best confirmed response to first documentation of PD) were analysed *post hoc* in subgroups defined by depth of response and by time‐to‐best confirmed response. “Early” and “late” responders were defined by time‐to‐best confirmed response of 0–4 months and >4 months, respectively. Patients in either subgroup could have recorded an initial response prior to their best response.

As in the main study [17], Kaplan–Meier methodology was used to estimate time‐to‐event survival characteristics, with stratified log‐rank tests and Cox models used for interarm comparisons of the time‐to‐event endpoints. To control for the possible guarantee‐time bias in the PFS analysis and to eliminate potential bias due to transient responses in the DOR analysis, sensitivity analyses were conducted in patients with a PFS or DOR of ≥6 months.

Multivariable analyses were conducted for IRd‐treated patients achieving partial response (PR) or better to identify any associations between PFS and the timing of response after controlling for potentially confounding baseline covariates. Analyses were performed using stratified log‐rank tests and Cox proportional hazard modelling. In addition to the randomisation stratification factors, other baseline covariates were considered in the model including, but not limited to, age, ISS stage at screening, BPI‐SF worst pain score, Eastern Cooperative Oncology Group performance status, CrCl, cytogenetic status, presence of amp(1q21), extramedullary disease at initial diagnosis, and lactate dehydrogenase (LDH) levels.

## 3 RESULTS

### 3.1 Best confirmed response in the intent‐to‐treat population

In total, 705 patients in the intent‐to‐treat (ITT) population underwent an assessment of IRC‐assessed best confirmed response: 20% had a CR or stringent CR (sCR), 35% achieved a VGPR, 26% had a PR, 10% had stable disease (SD), and 3% had PD. Best‐confirmed responses, overall and by treatment arm, are shown in Table 1.

**TABLE 1:** Best confirmed responses in the ITT population [17].

Response, %	Overall^a^ (*N* = 705)	IRd (*n* = 351)	Placebo‐Rd (*n* = 354)
CR (including sCR)	20	26	14
VGPR	35	37	34
PR	26	19	32
SD	10	9	10
PD	3	1	4
Not evaluable	7	8	6

Abbreviations: CR, complete response; IRd, ixazomib‐lenalidomide‐dexamethasone; ITT, intent‐to‐treat; PD, progressive disease; PR, partial response; Rd, lenalidomide‐dexamethasone; sCR, stringent complete response; SD, stable disease; VGPR, very good partial response.

^a^ Total may not sum to 100 due to rounding.

### 3.2 PFS and DOR by depth of best confirmed response

**FIGURE 1:**
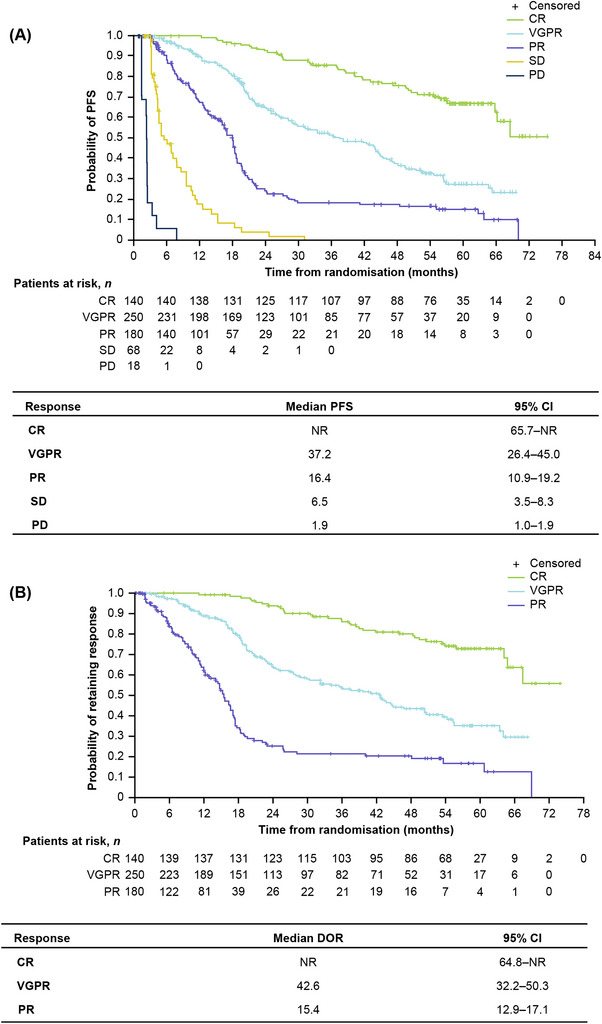
(A) PFS and (B) DOR pooled across IRd and placebo‐Rd arms by depth of best achieved response. CI, confidence interval; CR, complete response; DOR, duration of response; IRd, ixazomib‐lenalidomide‐dexamethasone; NR, not reached; PD, progressive disease; PFS; progression‐free survival; PR, partial response; Rd, lenalidomide‐dexamethasone; SD, stable disease; VGPR, very good partial response.

In a pooled analysis of patients from both treatment arms, achieving a deeper response was associated with improved PFS and a longer DOR. Median PFS was not reached (NR), 37.2, and 16.4 months for patients with CR, VGPR, and PR, respectively (Figure 1A). Median DOR was NR, 42.6, and 15.4 months for patients with CR, VGPR, and PR, respectively (Figure 1B).

### 3.3 Early versus late responders

**FIGURE 2:**
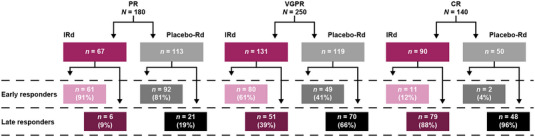
Early and late responders receiving IRd and placebo‐Rd who achieved PR, VGPR, or CR. CR, complete response; IRd, ixazomib‐lenalidomide‐dexamethasone; PR, partial response; Rd, lenalidomide‐dexamethasone; VGPR, very good partial response.

Among all 705 patients, 424 (60%) were defined as early responders and 281 (40%) were defined as late responders. In total, 570 patients receiving IRd (*n* = 288) or placebo‐Rd (*n* = 282) achieved PR or better (≥PR). Among patients achieving a PR (*n* = 180; IRd, *n* = 67; placebo‐Rd, *n* = 113), 85% (*n* = 153) were early responders and 15% (*n* = 27) were late responders. A total of 250 patients receiving IRd (*n* = 131) or placebo‐Rd (*n* = 119) achieved a VGPR; of these, 52% (*n* = 129) were early responders and 48% (*n* = 121) were late responders. A further 140 patients receiving IRd (*n* = 90) or placebo‐Rd (*n* = 50) achieved a CR; among these, a notably higher proportion were late responders (*n* = 117; 91%) versus early responders (*n* = 13; 9%) (Figure 2).

Patient demographics and baseline characteristics were generally similar between early versus late responders, although there was an imbalance in ISS stage (stage III, 21% vs. 12%), CrCl (CrCl ≤60 mL/minute, 46% vs. 37%), cytogenetics (expanded high‐risk cytogenetic abnormalities, 44% vs. 33%; presence of amp(1q21), 34% vs. 24%), and LDH levels (high LDH, 14% vs. 5%) (Table 2). Baseline characteristics by treatment and time to best response are shown in Table S1.

**TABLE 2:** Baseline demographics and characteristics in early versus late responders.

Patients, *n* (%)	Early responders (*n* = 424)	Late responders (*n* = 281)	*p*‐Value^a^
Age			0.13
<75 years	229 (54)	168 (60)	
≥75 years	195 (46)	113 (40)	
Sex			0.45
Female	216 (51)	135 (48)	
Male	208 (49)	146 (52)	
Race			0.45
White	342 (81)	234 (83)	
Black or African American	12 (3)	12 (4)	
Asian^b^	63 (15)	33 (12)	
Other^c^	7 (2)	2 (<1)	
ISS stage at screening			0.004
I or II	336 (79)	246 (88)	
III	88 (21)	35 (12)	
BPI‐SF worst pain rating at screening			0.53
<4	192 (45)	134 (48)	
≥4	232 (55)	147 (52)	
Baseline CrCl, mL/min			0.021
≤60	194 (46)	104 (37)	
>60	230 (54)	177 (63)	
Cytogenetics classification (molecular)			0.008
Expanded high‐risk^d^	187 (44)	93 (33)	
Standard risk	159 (38)	135 (48)	
Unclassified	78 (18)	53 (19)	
Cytogenetic abnormality^e^			
amp(1q)			0.007
Yes	143 (34)	68 (24)	
Unclassified or unknown	281 (66)	213 (76)	
del(17p)			0.079
Yes	47 (11)	20 (7)	
Unclassified or unknown	377 (89)	261 (93)	
t(4;14)			0.037
Yes	41 (10)	15 (5)	
Unclassified or unknown	383 (90)	266 (95)	
Type of myeloma at study entry^f^			0.43
IgA	81 (19)	61 (22)	
IgD	8 (2)	2 (<1)	
IgE	3 (<1)	0 (0)	
IgG	231 (54)	173 (62)	
IgM	2 (<1)	1 (<1)	
Biclonal	11 (3)	12 (4)	
Baseline LDH			<0.001
Normal	355 (84)	256 (91)	
Low	9 (2)	10 (4)	
High	60 (14)	15 (5)	
Extramedullary disease at initial diagnosis			0.40
No	383 (90)	260 (93)	
Yes	31 (7)	18 (6)	
Unknown	10 (2)	3 (1)	

Abbreviations: BPI‐SF, Brief Pain Inventory‐Short Form; CrCl, creatinine clearance; Ig, immunoglobuline; ISS, International Staging System; LDH, lactate dehydrogenase; PR, partial response.

^a^ Determined by Pearson's chi‐squared test, Wilcoxon rank sum test, or Fisher's exact test depending on the variable. *p*‐Values were calculated to identify possible covariates to include in the Cox proportional hazards model; however, they should be interpreted with caution as the analysis is *post hoc* and does not adjust for multiplicity.

^b^ Asian Indian, Chinese, Filipino, Japanese, Korean, Vietnamese.

^c^ American Indian or Alaskan Native, Native Hawaiian or other Pacific Islander, Other.

^d^ Includes t(4 ;14), t(14 ;16), del(17p), amp(1q21).

^e^ Six early responders and five late responders carried the t(14;16) cytogenetic abnormality.

^f^ Totals do not sum due to 88 and 32 missing early responder and later responder patients, respectively.

### 3.4 PFS and DOR in early and late responders

Median PFS with IRd was prolonged among late (65.7 months) versus early responders (21.2 months) in patients achieving ≥PR (HR 0.29; 95% CI: 0.20–0.42; *p* < 0.0001). With placebo‐Rd, median PFS was 62.6 versus 18.2 months for late versus early responders, respectively (HR 0.28; 95% CI: 0.20–0.40; *p* < 0.0001) (Figure S2A). Among patients achieving ≥VGPR, median PFS was also extended for late versus early responders, with respective median values of 65.7 versus 28.5 months with IRd (HR 0.38; 95% CI: 0.24–0.58; *p* < 0.0001) and 64.4 versus 21.6 months with placebo‐Rd (HR 0.35; 95% CI: 0.22–0.56; *p* < 0.0001) (Figure S2B). Longer median PFS among late versus early responders was observed regardless of treatment in patients achieving ≥PR or ≥VGPR (Figure S3A,B). Among patients achieving ≥CR, regardless of treatment, median PFS was not estimable for early or late responders (Figure S3C).

Similarly, median DOR was longer among late versus early responders with IRd and placebo‐Rd in patients who achieved ≥PR and ≥VGPR. Median DOR for late versus early responders was NR versus 22.6 months with IRd (HR 0.31; 95% CI: 0.21–0.46; *p* < 0.0001), and 64.1 versus 17.2 months with placebo‐Rd (HR 0.27; 95% CI: 0.19–0.39; *p* < 0.0001), respectively, in patients achieving ≥PR (Figure S4A). Among patients with ≥VGPR, median DOR for late versus early responders was NR versus 42.8 months with IRd (HR 0.40; 95% CI: 0.25–0.64; *p* < 0.0001) and 64.2 versus 25.1 months with placebo‐Rd (HR 0.31; 95% CI: 0.18–0.51; *p* < 0.0001), respectively (Figure S4B).

Analyses of PFS and DOR in late and early responders receiving IRd versus placebo‐Rd are summarised in Table S2.

### 3.5 Sensitivity analyses

**FIGURE 3:**
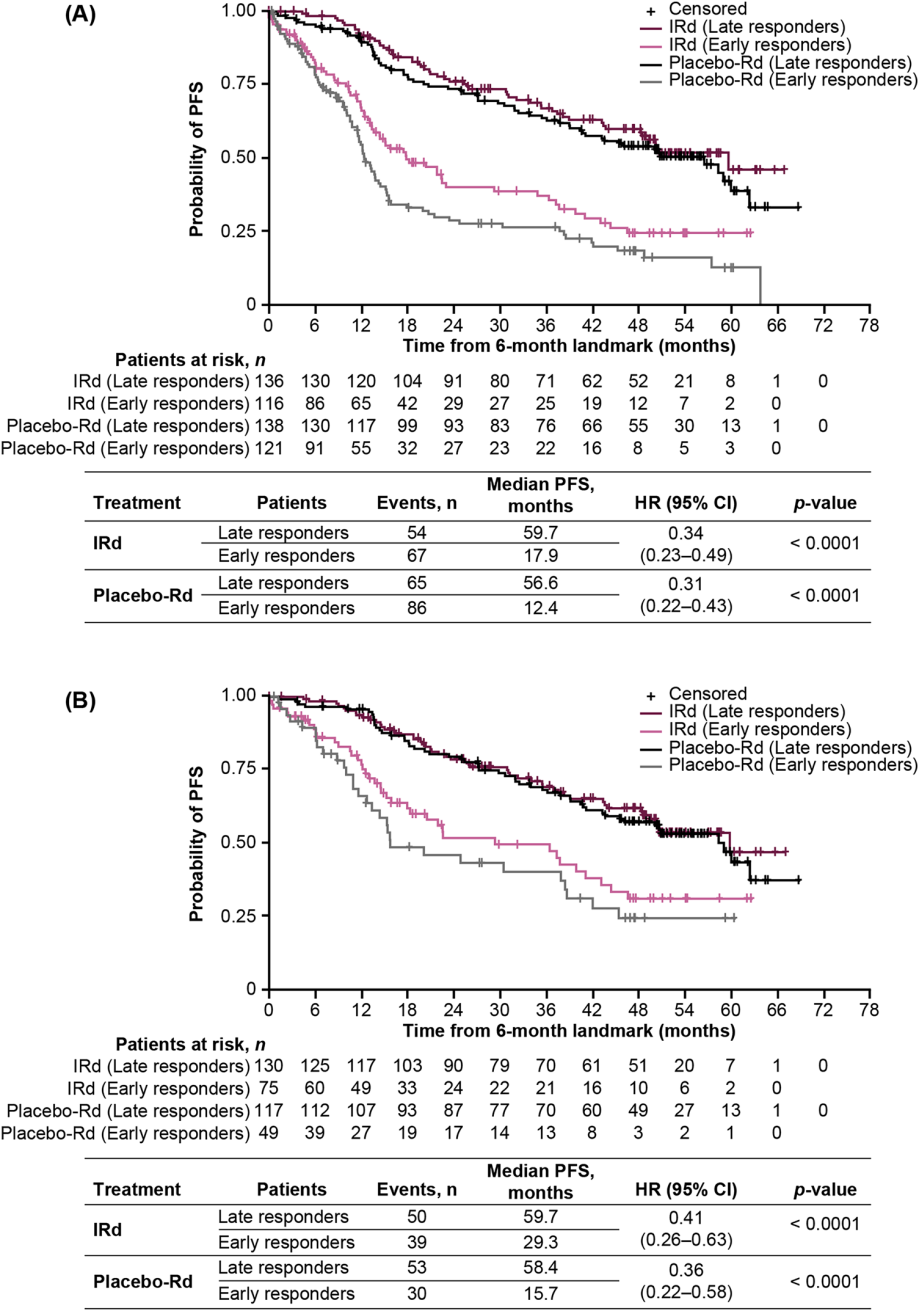
PFS lasting ≥6 months by treatment arm in early versus late responders achieving (A) ≥PR or (B) ≥VGPR. CI, confidence interval; HR, hazard ratio; IRd, ixazomib‐lenalidomide‐dexamethasone; PFS, progression‐free survival; PR, partial response; Rd, lenalidomide‐dexamethasone; VGPR, very good partial response.

The sensitivity analyses explored the relationship between time to response and outcomes among patients with a PFS or DOR of ≥6 months who achieved ≥PR or ≥VGPR. In patients with PFS lasting ≥6 months who achieved ≥PR (*n* = 511), median PFS was prolonged among late versus early responders with IRd (59.7 vs. 17.9 months; HR 0.34 95% CI: 0.23–0.49; *p* < 0.0001) and placebo‐Rd (56.6 vs. 12.4 months; HR 0.31 95% CI: 0.22–0.43; *p* < 0.0001) (Figure 3A). Similarly, in patients with PFS lasting ≥6 months who achieved ≥VGPR (*n* = 371), median PFS was prolonged among late versus early responders with IRd (59.7 vs. 29.3 months; HR 0.41; 95% CI: 0.26–0.63; *p* < 0.0001) and placebo‐Rd (58.4 vs. 15.7 months; HR 0.36; 95% CI: 0.22–0.58; *p* < 0.0001; Figure 3B).

**FIGURE 4:**
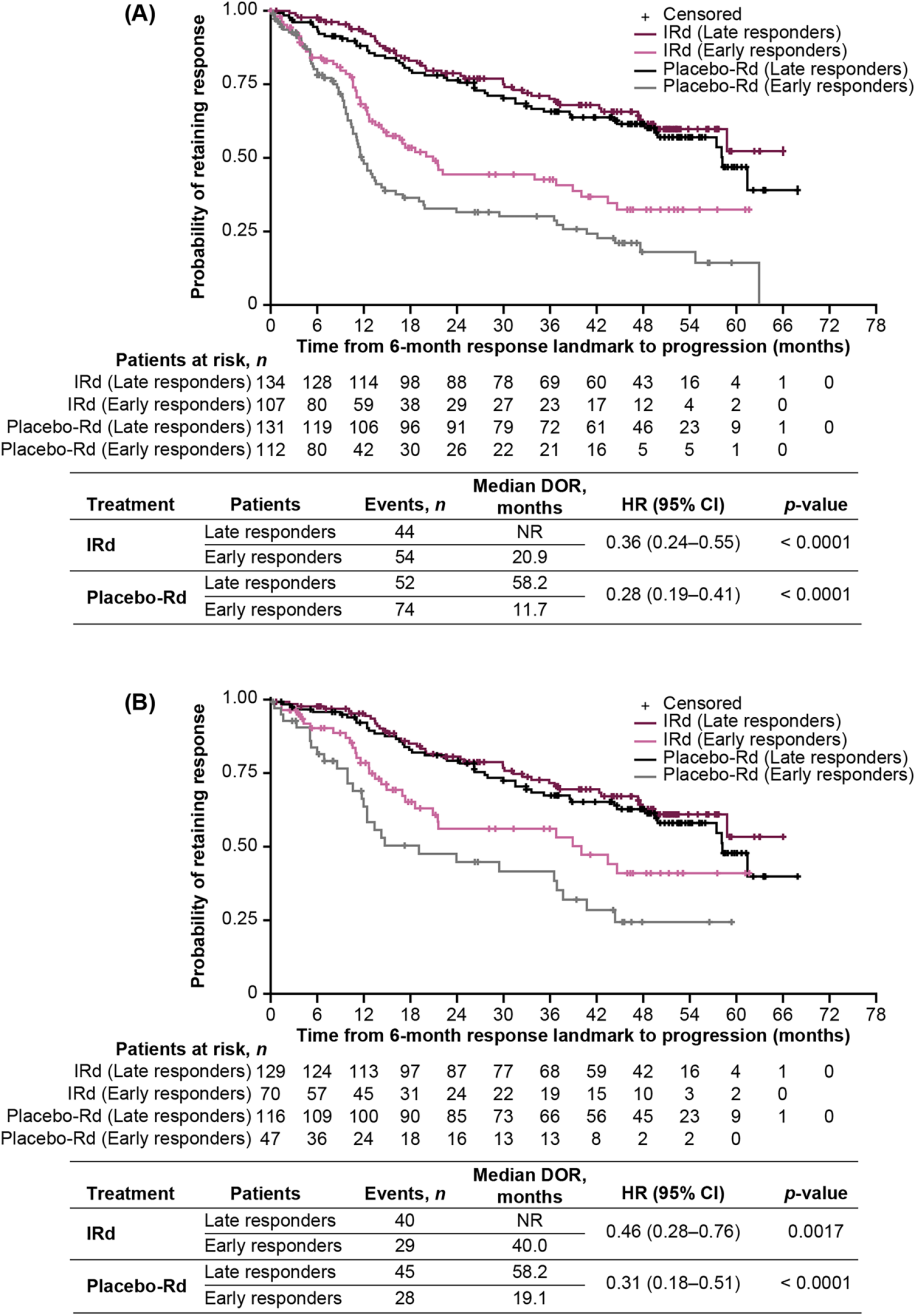
DOR lasting ≥6 months by treatment arm in early versus late responders achieving (A) ≥PR or (B) ≥VGPR. CI, confidence interval; DOR, duration of response; HR, hazard ratio; IRd, ixazomib‐lenalidomide‐dexamethasone; NR, not reached; PR, partial response; Rd, lenalidomide‐dexamethasone; VGPR, very good partial response.

In patients with DOR lasting ≥6 months who achieved ≥PR (*n* = 484), median DOR was prolonged among late versus early responders with IRd (NR vs. 20.9 months; HR 0.36 95% CI: 0.24–0.55; *p* < 0.0001) and placebo‐Rd (58.2 vs. 11.7 months; HR 0.28 95% CI: 0.19–0.41; *p* < 0.0001; Figure 4A). Similarly, in patients with DOR lasting ≥6 months achieving ≥VGPR (*n* = 362), median DOR was prolonged among late versus early responders with IRd (NR vs. 40.0 months; HR 0.46 95% CI: 0.28–0.76; *p* = 0.0017) and placebo‐Rd (58.2 vs. 19.1 months; HR 0.31 95% CI: 0.18–0.51; *p* < 0.0001; Figure 4B).

### 3.6 Multivariable analysis

Multivariable analyses by Cox proportional hazards modelling were conducted for IRd‐treated patients who achieved ≥PR to identify associations between PFS and the timing of response after controlling for baseline covariates. Randomisation stratification variables and baseline covariates characterising a difference between early and late responders were included in the adjusted full Cox proportional hazards model. Using early response as reference, a late response was significantly associated with longer PFS when adjusted for baseline covariates (HR 0.31; 95% CI: 0.21–0.46; *p* < 0.001; Figure S5). From the predictive model obtained from the multivariable analysis, achieving a late response remained independently associated with longer PFS (HR 0.31; 95% CI: 0.21–0.44; *p* < 0.001; Figure S6).

## 4 DISCUSSION

This post hoc analysis of patients with NDMM in TOURMALINE‐MM2 demonstrated that achieving a greater depth of response was associated with prolonged PFS and DOR. The PFS benefit with IRd versus placebo‐Rd on the ITT analysis appeared to be driven by higher rates of deep responses, with 63% versus 48% of patients achieving ≥VGPR. Moreover, the analysis of the effect of time to response on outcomes showed that PFS and DOR were also longer in patients achieving a late versus early best confirmed response of ≥PR or ≥VGPR, with a higher proportion of late responders achieving a deeper response (≥VGPR vs. ≥PR) than early responders.

Consistent with results from a similar analysis in RRMM in the TOURMALINE‐MM1 trial [15], our findings indicate that transplant‐ineligible patients with NDMM achieving a late ≥VGPR may have longer PFS and DOR than those achieving an earlier ≥VGPR. Since survival of late responders was guaranteed to be >4 months, PFS estimates may be biased towards late responders. Another potential bias is that achievement of a deeper response may typically take longer, and thus late responders would be enriched in deeper responses. To control for these potential biases, we conducted sensitivity analyses in patients with a PFS or DOR of ≥6 months who achieved ≥PR or ≥VGPR; the results confirmed the association of a late response with improved outcomes. Furthermore, in an analysis by treatment based on early versus late responders, our results demonstrate superior PFS for late responders in both arms, although it should also be noted that, in early responders, PFS was longer in patients who received IRd compared with those treated with placebo‐Rd (≥VGPR median PFS of 28.5 months vs. 21.6).

To explore potential biological differences between early and late responders, baseline characteristics were compared in an exploratory analysis. Interestingly, we observed differences in ISS stage, CrCl, cytogenetic risk, and LDH levels; patients who demonstrated high‐risk features according to these variables [19] were more likely to be in the early response group. While the comparison of baseline variables was only exploratory, thus limiting any conclusions we can make, our results do support the hypothesis proposed by Garderet et al. [15] that an early response is associated with higher proliferative activity. Patients with indolent disease may be slower to respond to therapy but an early response, characterised by higher plasma cell proliferation rates, may lead to an initial increased sensitivity to treatment and, consequently higher rates of loss of response and poorer long‐term outcomes [15, 16]. Due to significant differences observed in several baseline characteristics, we performed a multivariate analysis to evaluate the impact of early versus late response on PFS after adjusting for potentially confounding factors. Overall, timing of response was an independent prognostic factor for PFS on multivariable analyses of IRd‐treated patients who achieved ≥PR, with late response associated with superior PFS.

Alongside depth of response, our findings indicate that a slow, gradual response may also serve as a powerful prognostic factor given that a longer PFS was achieved in patients who had obtained a response in ≥4 months versus <4 months. By assessing late responders for what drives this superior PFS, a higher proportion had deeper responses versus early responders. There is a clear association between deeper responses and improved outcomes, as demonstrated by the prolonged PFS in patients with measurable residual disease (MRD) negativity in a pooled analysis of two previous studies investigating ixazomib maintenance [20].

The relationship between MRD and response kinetics was not assessed in the current study, thus further analyses are needed to assess whether patients who respond later are more likely to achieve MRD negativity. Accordingly, our results demonstrate that slowness of response to treatment with a PI‐IMiD‐steroid combination is not a negative predictor of outcome for patients with NDMM. The potential of treatment adaptation according to response depth and duration has been acknowledged and may lead to a more tailored approach to therapeutic decision‐making [21].

The exploratory nature of this analysis is a limitation of the study as our results are inconclusive and require further confirmation. However, our conclusions do support previously reported findings [15] and contribute to the understanding of response kinetics in MM. Analyses of studies involving other classes of MM agents are needed to determine whether response kinetics impact outcomes in a similar manner for other therapies. Encouragingly, recent data on daratumumab‐based triplets in RRMM also report better outcomes (PFS and DOR) in late versus early responders [22], supporting the notion that patients without an early response can benefit from continued antimyeloma treatment. Due to the exploratory nature and complexity of the analyses, which are based on treatment until progression and analysis of best response achieved, it is difficult to apply the results clinically, and to understand the relative contributions of kinetics and depth of response.

In conclusion, our results confirm that a greater depth of response was associated with both prolonged PFS and DOR in transplant‐ineligible patients with NDMM. Importantly, we evaluated the relationship between rapidity of response and outcomes and observed that PFS and DOR were longer in patients achieving a late (≥4 months) versus early (<4 months) best confirmed response, and that a higher proportion of late responders achieved a deeper response (CR vs. VGPR or PR) than early responders. Moreover, we demonstrated superior outcomes for late versus early responders in both treatment arms. Our findings thus indicate that a slow response to therapy may be a powerful prognostic factor and highlight that slowness of response to treatment with a PI‐IMiD‐steroid combination is not a negative predictor of outcome in NDMM.

## AUTHOR CONTRIBUTIONS

*Conception or design of the work*: Xioaquan Zhang, Philip Twumasi‐Ankrah, and Richard Labotka. *Acquisition, analysis, or interpretation of data*: Paul G. Richardson, Thierry Facon, Christopher P. Venner, Nizar J. Bahlis, Fritz Offner, Darrell White, Lionel Karlin, Lofti Benboubker, Eric Voog, Sung‐Soo Yoon, Kenshi Suzuki, Hirohiko Shibayama, Xiaoquan Zhang, Miguel Villarreal, Philip Twumasi‐Ankrah, Richard Labotka, Robert M. Rifkin, Sagar Lonial, Shaji K. Kumar, S. Vincent Rajkumar, and Philippe Moreau. All authors drafted and revised the work critically, approved the final version of the manuscript and agree to be accountable for all aspects of the work.

## CONFLICT OF INTEREST STATEMENT

Paul G. Richardson: Consultancy: BMS/Celgene, Takeda, GSK, Sanofi, Oncopeptides, Secura Bio, Karyopharm, AstraZeneca, Novartis; Research funding: Oncopeptides, BMS/Celgene, Takeda, Karyopharm. Nizar J. Bahlis: Honoraria and advisory board member: Karyopharm, Genentech, Janssen, BMS, Amgen, Takeda, AbbVie, GSK, Sanofi, Pfizer; Research funding: Pfizer. Darrell White: Honoraria: Amgen, Antengene, BMS/Celgene, FORUS Therapeutics, Sanofi, GSK, Janssen, Takeda, Karyopharm. Lionel Karlin: Honoraria: Janssen, AbbVie, Amgen, GSK, Sanofi, BMS/Celgene, Takeda; Advisory board member: Janssen, Amgen, GSK, BMS/Celgene, Takeda, Sanofi. Sung‐Soo Yoon: Member of advisory board: AbbVie, Amgen, Janssen, Novartis, Regeneron; Research funding: Roche‐Genentech, Kyowa‐Kirin, Yuhan Pharma. Christopher P. Venner: Honoraria: Takeda, BMS, Janssen, Pfizer, Sanofi, GSK, AbbVie, FORUS Therapeutics. Kenshi Suzuki: Consultancy: Amgen, Takeda, BMS; Research funding: BMS; Honoraria: BMS, Amgen, Takeda, ONO, Novartis, Sanofi, AbbVie, Janssen. Hirohiko Shibayama: Research funding: Celgene, Ono, AbbVie, Eisai, Novartis, Janssen, Chugai, Essential Pharma Japan, Sanofi, AstraZeneca, HUYABIO International; Honoraria: Ono, Takeda, AbbVie, Eisai, Novartis, Janssen, Chugai, AstraZeneca, Sanofi, Celgene, SymBio, Kyowa Kirin. Xiaoquan Zhang, Miguel Villarreal, Philip Twumasi‐Ankrah: Employee: Takeda. Richard Labotka: Employee and equity holder: Takeda. Robert M. Rifkin: Member of board of directors/advisory committee: Amgen, BMS/Celgene, Coherus, Fresenius‐Kabi, Sanofi, Takeda, Karyopharm; Current employment and equity holder: McKesson. Sagar Lonial: Consultancy: Janssen, BMS/Celgene, Amgen, GSK, AbbVie, Takeda, Genentech, Pfizer, Regeneron; Research funding: Janssen, BMS/Celgene, GSK, Takeda; Honoraria: Janssen, BMS/Celgene, Amgen, GSK, Takeda, AbbVie, Merck; Member of board of directors/advisory committee: TG Therapeutics; Ownership of stock/shares: TG Therapeutics. Shaji K. Kumar: Research funding: Celgene, Adaptive, Sanofi, AbbVie, Takeda, Janssen, KITE, Merck, MedImmune/AstraZeneca, Novartis, Roche; Advisory board member: AbbVie, Celgene, Janssen, Takeda, Adaptive, KITE, MedImmune/AstraZeneca; Member of independent review committee: Oncopeptides. Philippe Moreau: Consultancy and member of advisory board: Janssen, Celgene, Takeda, Amgen, Sanofi, AbbVie, GSK. Thierry Facon, Fritz Offner, Lotfi Benboubker, Eric Voog, and S. Vincent Rajkumar have no conflict of interest to declare.

## FUNDING INFORMATION

Millennium Pharmaceuticals, Inc., Cambridge, MA, USA, a wholly owned subsidiary of Takeda Pharmaceutical Company Limited.

## ETHICS STATEMENT

The trial was conducted in accordance with the International Conference on Harmonization Good Clinical Practice guideline and appropriate regulatory requirements. Local ethics committees or institutional review boards approved the protocol.

## PATIENT CONSENT STATEMENT

All patients provided written informed consent.

## PERMISSIONS STATEMENT

Table 1, Table 2, Figure 1A, and Figure 3 are from: Richardson PG, et al. ASH 2021; poster 2733. This abstract was originally published in *Blood*. Richardson, PG. et al. Late versus Early Response and Depth of Response Are Associated with Improved Outcomes in Newly Diagnosed Multiple Myeloma (NDMM) Patients (pts) Treated with Ixazomib‐Lenalidomide‐Dexamethasone (IRd) or Placebo‐Lenalidomide‐Dexamethasone (pbo‐Rd) in the Phase 3 TOURMALINE‐MM2 Trial. *Blood* (2021) 138 (Supplement 1): 2733, © the American Society of Hematology. Reproduced with permission from the author.

## CLINICAL TRIAL REGISTRATION

Clinicaltrials.gov, NCT01850524

## Supporting information

Supporting Information

## Data Availability

The datasets, including the redacted study protocol, redacted statistical analysis plan, and individual participants’ data supporting the results reported in this article, will be made available within 3 months from initial request to researchers who provide a methodologically sound proposal. The data will be provided after its de‐identification, in compliance with applicable privacy laws, data protection, and requirements for consent and anonymization.
