# Supplementary material for: Late versus early response and depth of response are associated with improved outcomes in patients with newly diagnosed multiple myeloma enrolled in the TOURMALINE‐MM2 trial
Source: EJHaem. 2023 Aug 3;4(4):995–1005. doi: 10.1002/jha2.759 (PMC10660432; doi:10.1002/jha2.759)
Supplement: Supplementary file 1 — Supporting Information [file JHA2-4-995-s001.docx]

# **SUPPORTING INFORMATION**

**SUPPLEMENTARY TABLES**

**TABLE S1** Patient demographics and baseline characteristics by treatment arm and timing of response in patients achieving ≥PR as best response during treatment

|  | **IRd** | | | **Placebo-Rd** | | |
| --- | --- | --- | --- | --- | --- | --- |
| Patients | **Early responders (*n* = 152)** | **Late responders**  **(*n* = 136)** | ***p*-value^a^** | **Early responders (*n* = 143)** | **Late responders (*n* = 139)** | ***p*-value^a^** |
| Age, *n* (%) |  |  | 0.45 |  |  | 0.38 |
| <75 years | 85 (56) | 82 (60) |  | 78 (55) | 83 (60) |  |
| ≥75 years | 67 (44) | 54 (40) |  | 65 (45) | 56 (40) |  |
| Sex, *n* (%) |  |  | 0.79 |  |  | 0.23 |
| Female | 77 (51) | 71 (52) |  | 73 (51) | 61 (44) |  |
| Male | 75 (49) | 65 (48) |  | 70 (49) | 78 (56) |  |
| Race, *n* (%) |  |  | 0.99 |  |  | 0.001 |
| White | 127 (84) | 113 (83) |  | 110 (77) | 117 (84) |  |
| Black or African American | 5 (3) | 5 (4) |  | 1 (<1) | 7 (5) |  |
| Asian^b^ | 17 (11) | 18 (13) |  | 31 (22) | 13 (9) |  |
| Other^c^ | 3 (2) | 0 (0) |  | 1 (<1) | 2 (1) |  |
| Baseline ECOG Performance Status, *n* (%) |  |  | 0.011 |  |  | 0.83 |
| 0 | 43 (28) | 51 (38) |  | 49 (34) | 47 (34) |  |
| 1 | 77 (51) | 73 (54) |  | 74 (52) | 69 (50) |  |
| 2 | 32 (21) | 12 (9) |  | 20 (14) | 23 (17) |  |
| ISS stage at screening, *n* (%) |  |  | 0.54 |  |  | 0.044 |
| I or II | 128 (84) | 118 (87) |  | 114 (80) | 123 (88) |  |
| III | 24 (16) | 18 (13) |  | 29 (20) | 16 (12) |  |
| BPI-SF worst pain rating at screening, *n* (%) |  |  | 0.30 |  |  | 0.47 |
| <4 | 69 (45) | 70 (51) |  | 71 (50) | 63 (45) |  |
| ≥4 | 83 (55) | 66 (49) |  | 72 (50) | 76 (55) |  |
| Baseline weight, kg |  |  | 0.002 |  |  | 0.75 |
| Mean (StDev) | 76 (18) | 69 (14) |  | 70 (16) | 70 (14) |  |
| Median (IQR) | 75 (63–88) | 68 (59–78) |  | 69 (59–80) | 70 (61–78) |  |
| Range | 40–127 | 42–114 |  | 34–124 | 38–115 |  |
| Baseline serum albumin, g/dL |  |  | 0.41 |  |  | 0.065 |
| Mean (StDev) | 4 (1) | 4 (1) |  | 4 (1) | 4 (1) |  |
| Median (IQR) | 4 (4–4) | 4 (4–4) |  | 4 (3–4) | 4 (4–4) |  |
| Range | 2–5 | 2–5 |  | 2–5 | 2–5 |  |
| Baseline CrCl, n (%) |  |  | 0.98 |  |  | 0.061 |
| ≤60 mL/min | 59 (39) | 53 (39) |  | 65 (45) | 48 (35) |  |
| >60 mL/min | 93 (61) | 83 (61) |  | 78 (55) | 91 (65) |  |
| Baseline haemoglobin, mg/dL |  |  | 0.42 |  |  | 0.003 |
| Mean (StDev) | 105 (18) | 107 (17) |  | 102 (15) | 108 (18) |  |
| Median (IQR) | 102 (93–119) | 105 (94–118) |  | 102 (89–112) | 108 (93–121) |  |
| Range | 67–159 | 69–146 |  | 73–134 | 59–151 |  |
| Baseline FLC involved level, mg/L |  |  | 0.50 |  |  | 0.12 |
| Mean (StDev) | 1318 (2328) | 1156 (2270) |  | 1170 (2183) | 844 (1550) |  |
| Median (IQR) | 505 (93–1264) | 361 (101–1019) |  | 399 (136–1026) | 306 (84–858) |  |
| Range | 9–12 700 | 6–12 700 |  | 5–12 610 | 7–11 006 |  |
| Baseline urine M protein, mg/24hr |  |  | 0.66 |  |  | 0.27 |
| Mean (StDev) | 713 (1616) | 552 (1217) |  | 648 (1547) | 613 (2039) |  |
| Median (IQR) | 79 (0–539) | 79 (0–420) |  | 40 (0–405) | 38 (0–279) |  |
| Range | 0–13 171 | 0–8865 |  | 0–9942 | 0–15 676 |  |
| Baseline skeletal survey result, *n* (%) |  |  | 0.97 |  |  | 0.24 |
| Within normal limits | 19 (12) | 17 (12) |  | 22 (15) | 17 (12) |  |
| Abnormal, not clinically significant | 52 (34) | 43 (32) |  | 53 (37) | 39 (28) |  |
| Abnormal, clinically significant | 69 (45) | 64 (47) |  | 57 (40) | 70 (50) |  |
| Not done | 12 (8) | 12 (9) |  | 11 (8) | 13 (9) |  |
| Cytogenetics classification (molecular), *n* (%) |  |  | 0.011 |  |  | 0.008 |
| Expanded high-risk^d^ | 66 (43) | 46 (34) |  | 71 (50) | 44 (32) |  |
| Standard risk | 53 (35) | 71 (52) |  | 49 (34) | 62 (45) |  |
| Unclassified | 33 (22) | 19 (14) |  | 23 (16) | 33 (24) |  |
| Cytogenetic abnormality,^e^ *n* (%) |  |  |  |  |  |  |
| amp(1q) |  |  | 0.013 |  |  | 0.040 |
| Yes | 55 (36) | 31 (23) |  | 51 (36) | 34 (24) |  |
| Unclassified or unknown | 97 (64) | 105 (77) |  | 92 (64) | 105 (76) |  |
| del(17p) |  |  | 0.32 |  |  | 0.13 |
| Yes | 15 (10) | 9 (7) |  | 18 (13) | 10 (7) |  |
| Unclassified or unknown | 137 (90) | 127 (93) |  | 125 (87) | 129 (93) |  |
| t(4;14) |  |  | 0.21 |  |  | 0.088 |
| Yes | 15 (10) | 8 (6) |  | 15 (10) | 7 (5) |  |
| Unclassified or unknown | 137 (90) | 128 (94) |  | 128 (90) | 132 (95) |  |
| Type of myeloma at study entry,^f^ *n* (%) |  |  | 0.26 |  |  | 0.47 |
| IgA | 27 (18) | 33 (24) |  | 34 (24) | 27 (19) |  |
| IgD | 4 (3) | 1 (1) |  | 1 (1) | 1 (1) |  |
| IgE | 2 (1) | 0 (0) |  | 1 (1) | 0 (0) |  |
| IgG | 71 (47) | 82 (60) |  | 87 (61) | 88 (63) |  |
| IgM | 2 (1) | 0 (0) |  | 0 (0) | 1 (1) |  |
| Biclonal | 6 (4) | 5 (4) |  | 3 (2) | 7 (5) |  |
| Baseline LDH, *n* (%) |  |  | < 0.001 |  |  | 0.86 |
| Normal | 124 (82) | 126 (93) |  | 128 (90) | 125 (90) |  |
| Low | 2 (1) | 6 (4) |  | 3 (2) | 4 (3) |  |
| High | 26 (17) | 4 (3) |  | 12 (8) | 10 (7) |  |
| Extramedullary disease at initial diagnosis, *n* (%) |  |  | 0.063 |  |  | 0.31 |
| Yes | 13 (9) | 4 (3) |  | 7 (5) | 13 (9) |  |
| No | 135 (89) | 131 (96) |  | 132 (92) | 124 (89) |  |
| Unknown | 4 (3) | 1 (1) |  | 4 (3) | 2 (1) |  |

Abbreviations: BPI-SF, Brief Pain Inventory-Short Form; CrCl, creatinine clearance; ECOG, Eastern Cooperative Oncology Group; FLC, free light chain; Ig, immunoglobulin; IQR, interquartile range; ISS, International Staging System; IRd, ixazomib-lenalidomide-dexamethasone; LDH, lactate dehydrogenase; PR, partial response; Rd, lenalidomide-dexamethasone; StDev, standard deviation.

^a^Determined by Fisher’s exact test, Pearson’s chi-squared test, or Wilcoxon rank sum test depending on the variable. p-values were calculated to identify possible covariates to include in the Cox proportional hazards model; however, they should be interpreted with caution as the analysis is *post hoc* and does not adjust for multiplicity.

^b^Asian Indian, Chinese, Filipino, Japanese, Korean, Vietnamese.

^c^American Indian or Alaskan Native, Native Hawaiian or other Pacific Islander, Other.

^d^Includes t(4;14), t(14;16), del(17p), amp(1q21).

**^e^**Six early responders and 5 late responders carried the t(14;16) cytogenetic abnormality.

^f^Totals do not sum due to 36 and 15 missing early responder and late responder patients, respectively, who received IRd, and, 17 and 15 missing early responder and late responder patients, respectively, who received placebo-Rd.

**TABLE S2** PFS and DOR by time-to-best confirmed response in patients receiving IRd versus placebo-Rd

| **Patients** | **Treatment** | **Events** | **Median PFS/DOR, months** | **HR (95% CI)** | ***p*-value** |
| --- | --- | --- | --- | --- | --- |
| PFS by time of response in patients receiving IRd versus placebo-Rd achieving ≥PR | | | | | |
| Late responders | IRd | 54 | 65.7 | 0.88 (0.61–1.26) | 0.4796 |
|  | Placebo-Rd | 65 | 62.6 |  |  |
| Early responders | IRd | 83 | 21.2 | 0.84 (0.62–1.13) | 0.2564 |
|  | Placebo-Rd | 97 | 18.2 |  |  |
| PFS by time of response in patients receiving IRd versus placebo-Rd achieving ≥VGPR | | | | | |
| Late responders | IRd | 50 | 65.7 | 0.95 (0.64–1.41) | 0.8131 |
|  | Placebo-Rd | 53 | 64.4 |  |  |
| Early responders | IRd | 43 | 28.5 | 0.85 (0.53–1.37) | 0.5037 |
|  | Placebo-Rd | 31 | 21.6 |  |  |
| DOR by time of response in patients receiving IRd versus placebo-Rd achieving ≥PR | | | | | |
| Late responders | IRd | 44 | NR | 0.83 (0.55–1.24) | 0.3594 |
|  | Placebo-Rd | 55 | 64.1 |  |  |
| Early responders | IRd | 69 | 22.6 | 0.77 (0.55–1.06) | 0.1048 |
|  | Placebo-Rd | 86 | 17.2 |  |  |
| DOR by time of response in patients receiving IRd versus placebo-Rd achieving ≥VGPR | | | | | |
| Late responders | IRd | 40 | NR | 0.89 (0.58–1.38) | 0.6053 |
|  | Placebo-Rd | 45 | 64.2 |  |  |
| Early responders | IRd | 35 | 42.8 | 0.75 (0.45–1.24) | 0.2603 |
|  | Placebo-Rd | 28 | 25.1 |  |  |
| PFS lasting ≥6 months by time of response in patients receiving IRd versus placebo-Rd achieving ≥PR | | | | | |
| Late responders | IRd | 54 | 59.7 | 0.88 (0.61–1.26) | 0.4796 |
|  | Placebo-Rd | 65 | 56.6 |  |  |
| Early responders | IRd | 67 | 17.9 | 0.75 (0.54–1.04 | 0.0873 |
|  | Placebo-Rd | 86 | 12.4 |  |  |
| PFS lasting ≥6 months by time of response in patients receiving IRd versus placebo-Rd achieving ≥VGPR | | | | | |
| Late responders | IRd | 50 | 59.7 | 0.95 (0.64–1.41) | 0.8131 |
|  | Placebo-Rd | 53 | 58.4 |  |  |
| Early responders | IRd | 39 | 29.3 | 0.78 (0.47–1.27) | 0.3122 |
|  | Placebo-Rd | 30 | 15.7 |  |  |
| DOR lasting ≥6 months by time of response in patients receiving IRd versus placebo-Rd achieving ≥PR | | | | | |
| Late responders | IRd | 44 | NR | 0.88 (0.59–1.32) | 0.5372 |
|  | Placebo-Rd | 52 | 58.2 |  |  |
| Early responders | IRd | 54 | 20.9 | 0.69 (0.48–0.98) | 0.0376 |
|  | Placebo-Rd | 74 | 11.7 |  |  |
| DOR lasting ≥6 months by time of response in patients receiving IRd versus placebo-Rd achieving ≥VGPR | | | | | |
| Late responders | IRd | 40 | NR | 0.89 (0.58–1.38) | 0.6053 |
|  | Placebo-Rd | 45 | 58.2 |  |  |
| Early responders | IRd | 29 | 40.0 | 0.61 (0.36–1.05) | 0.0719 |
|  | Placebo-Rd | 28 | 19.1 |  |  |

Abbreviations: CI, confidence interval; DOR, duration of response; HR, hazard ratio; IRd, ixazomib-lenalidomide-dexamethasone; NR, not reached; PFS, progression-free survival; PR, partial response; Rd, lenalidomide-dexamethasone; VGPR, very good partial response.

**SUPPLEMENTARY FIGURES**

**FIGURE S1** TOURMALINE-MM2 study design.^1^


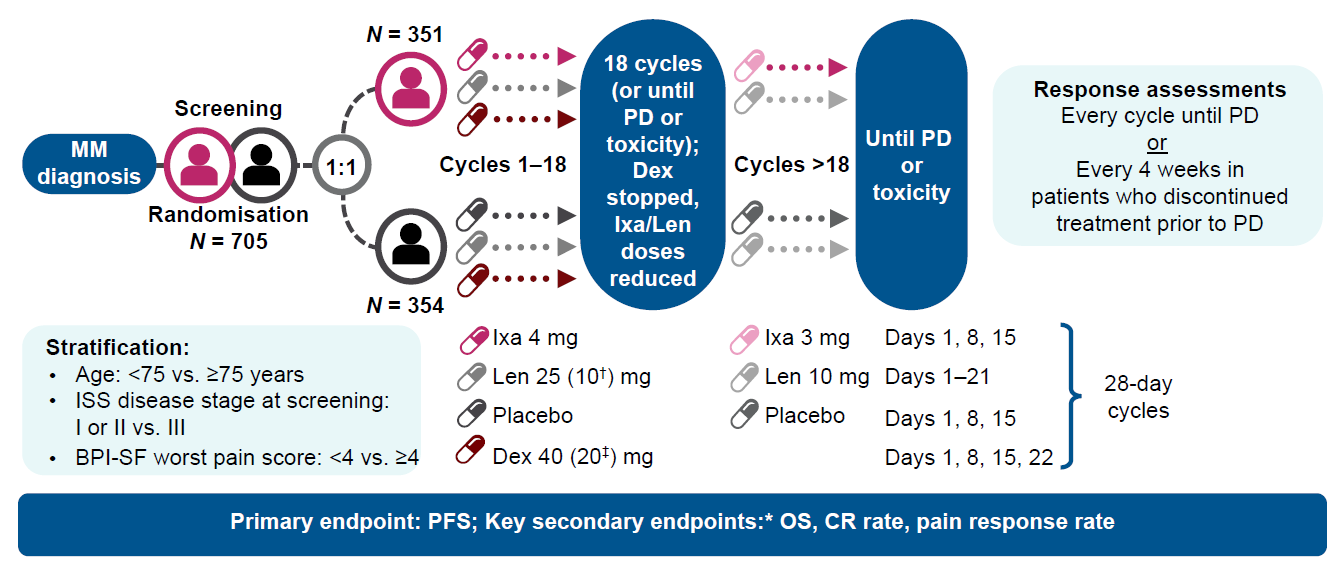


*Additional secondary endpoints included time to progression and safety.

^†^Patients with renal impairment.

^‡^Patients aged >75 years.

BPI-SF, Brief Pain Inventory-Short Form; CR, complete response; Dex, dexamethasone; ISS, International Staging System; Ixa, ixazomib; Len, lenalidomide; MM, multiple myeloma; OS, overall survival; PD, progressive disease; PFS, progression-free survival.

**Figure S2** PFS by treatment arm in early versus late responders achieving (A) ≥PR or (B) ≥VGPR.

**(A)**


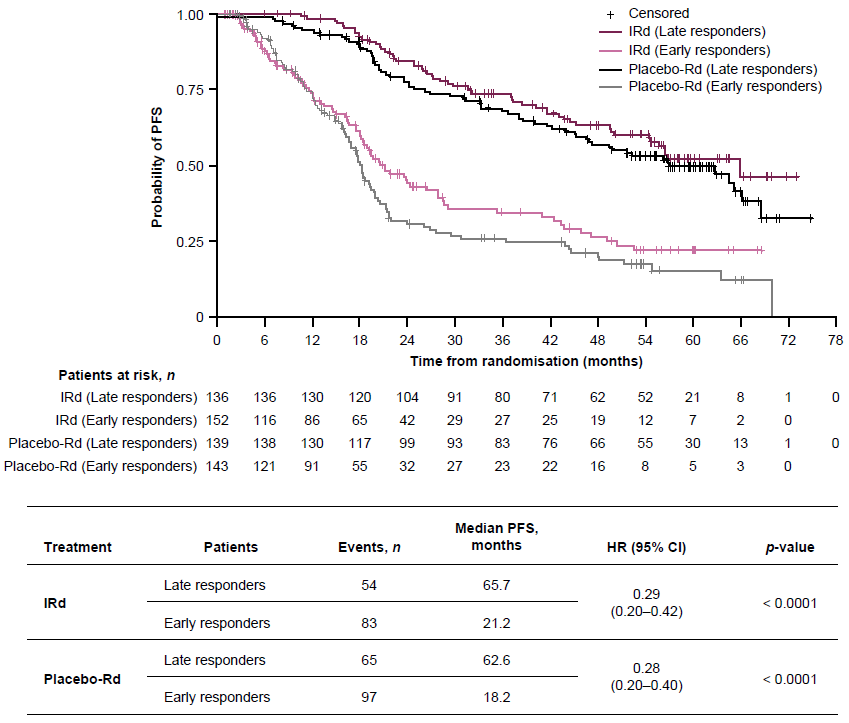


**(B)**


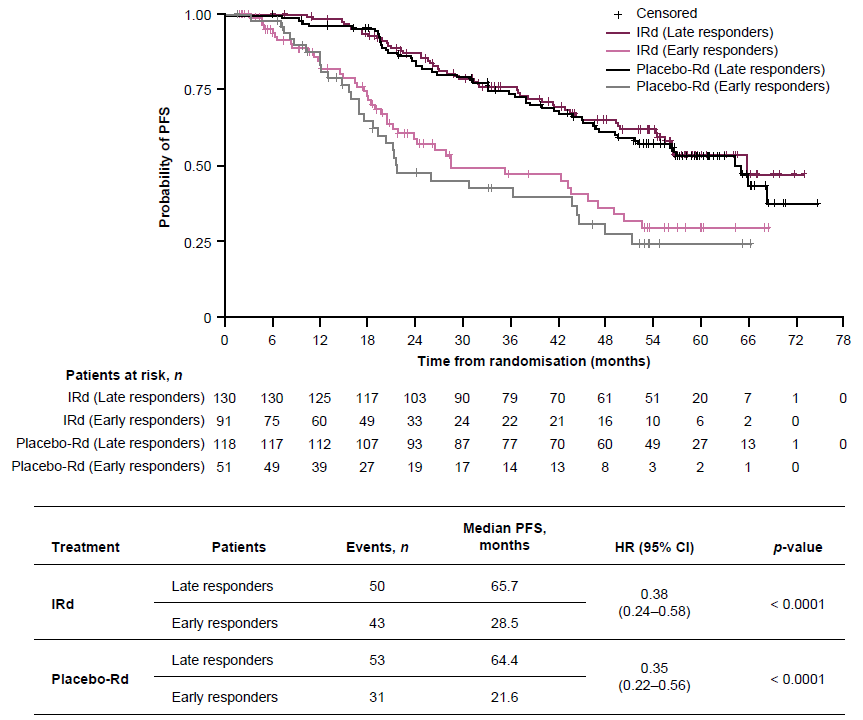


CI, confidence interval; HR, hazard ratio; IRd, ixazomib-lenalidomide-dexamethasone; PFS, progression-free survival; PR, partial response; Rd, lenalidomide-dexamethasone; VGPR, very good partial response.

**FIGURE S3** PFS in early versus late responders achieving (A) ≥PR, (B) ≥VGPR, or (C) ≥CR regardless of treatment.

**(A)**


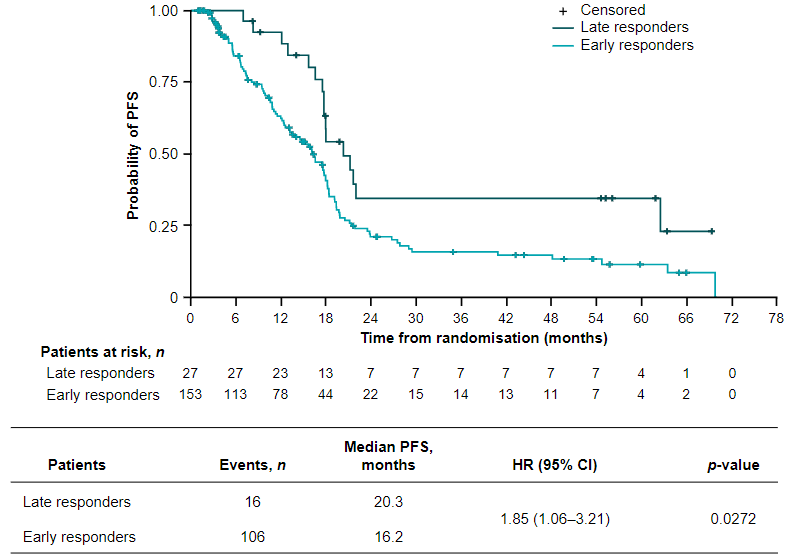


**(B)**


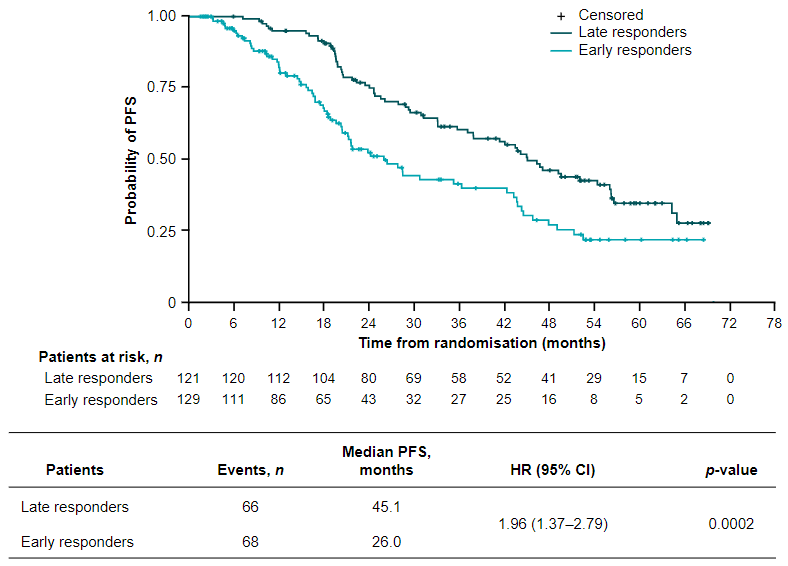


**(C)**


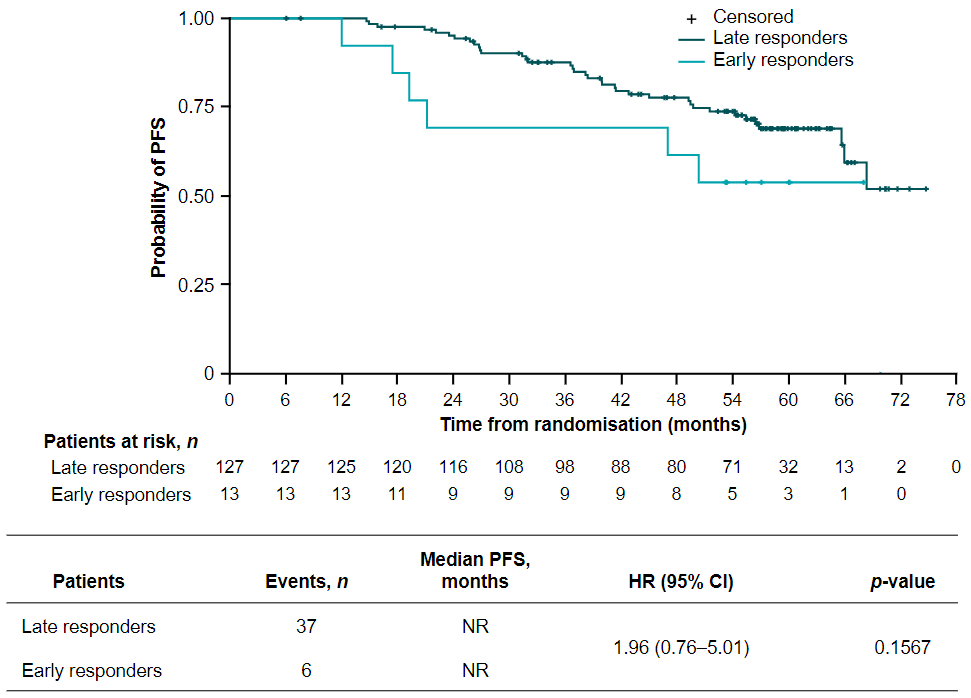


CI, confidence interval; CR, complete response; HR, hazard ratio; NR, not reached; PFS, progression-free survival; PR, partial response; VGPR, very good partial response.

**Figure S4** DOR by treatment arm in early versus late responders achieving (A) ≥PR and (B) ≥VGPR.

**(A)**


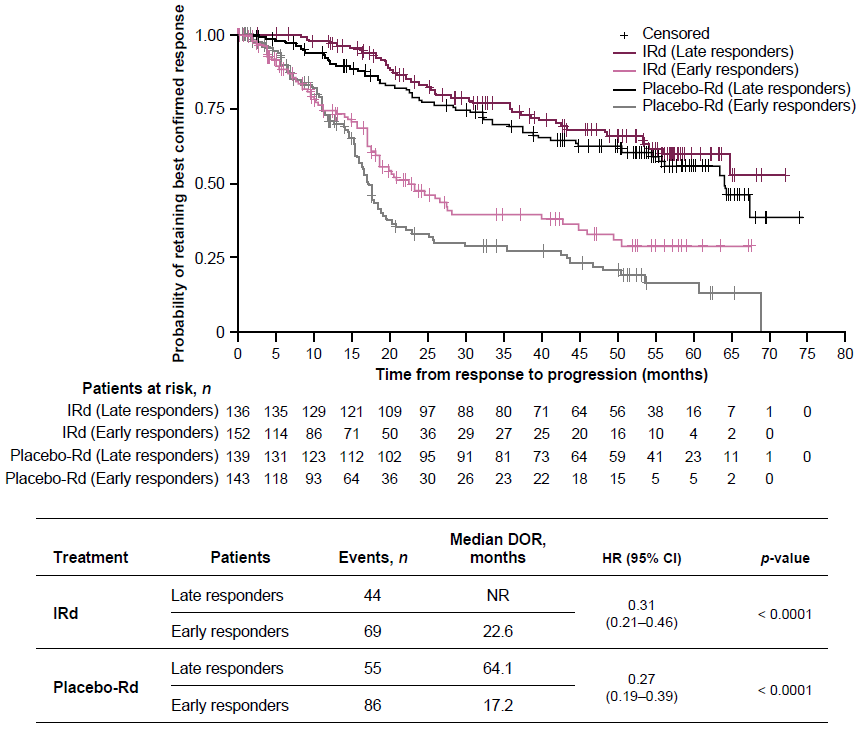


**(B)**


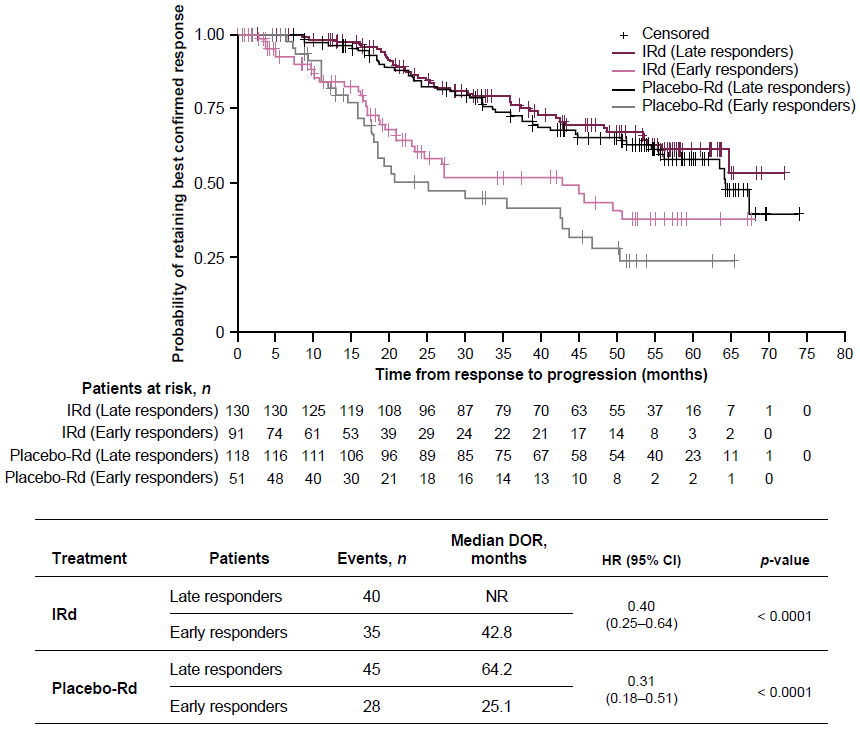


CI, confidence interval; DOR, duration of response; HR, hazard ratio; IRd, ixazomib-lenalidomide-dexamethasone; NR, not reached; PR, partial response; Rd, lenalidomide-dexamethasone; VGPR, very good partial response.

**FIGURE S5** Multivariable analysis of PFS for late versus early responders achieving ≥PR (full model).


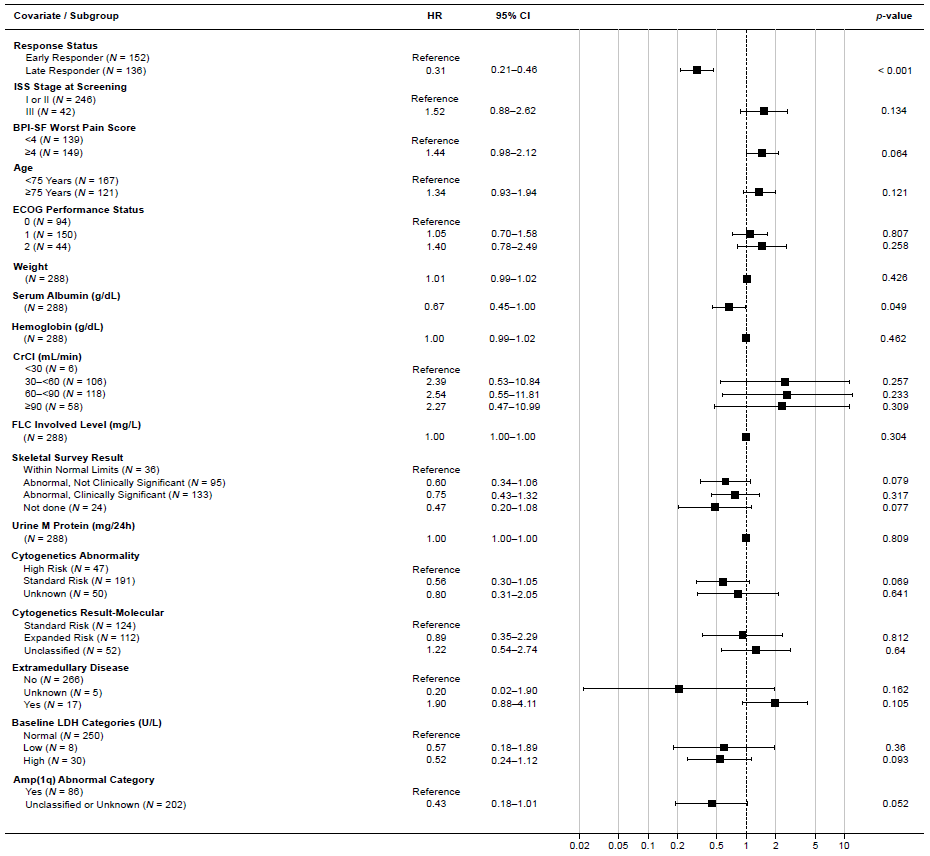


Cox proportional hazards model were used for HR values comparing baseline covariates of interest between late and early responders.

BPI-SF, Brief Pain Inventory-Short Form; CI, confidence interval; CrCl, creatinine clearance; ECOG, Eastern Cooperative Oncology Group; FLC, free light chain; HR, hazard ratio; ISS, International Staging System; LDH, lactate dehydrogenase; PFS, progression-free survival; PR, partial response.

**FIGURE S6** Multivariable analysis of PFS for late versus early responders achieving ≥PR (reduced model).


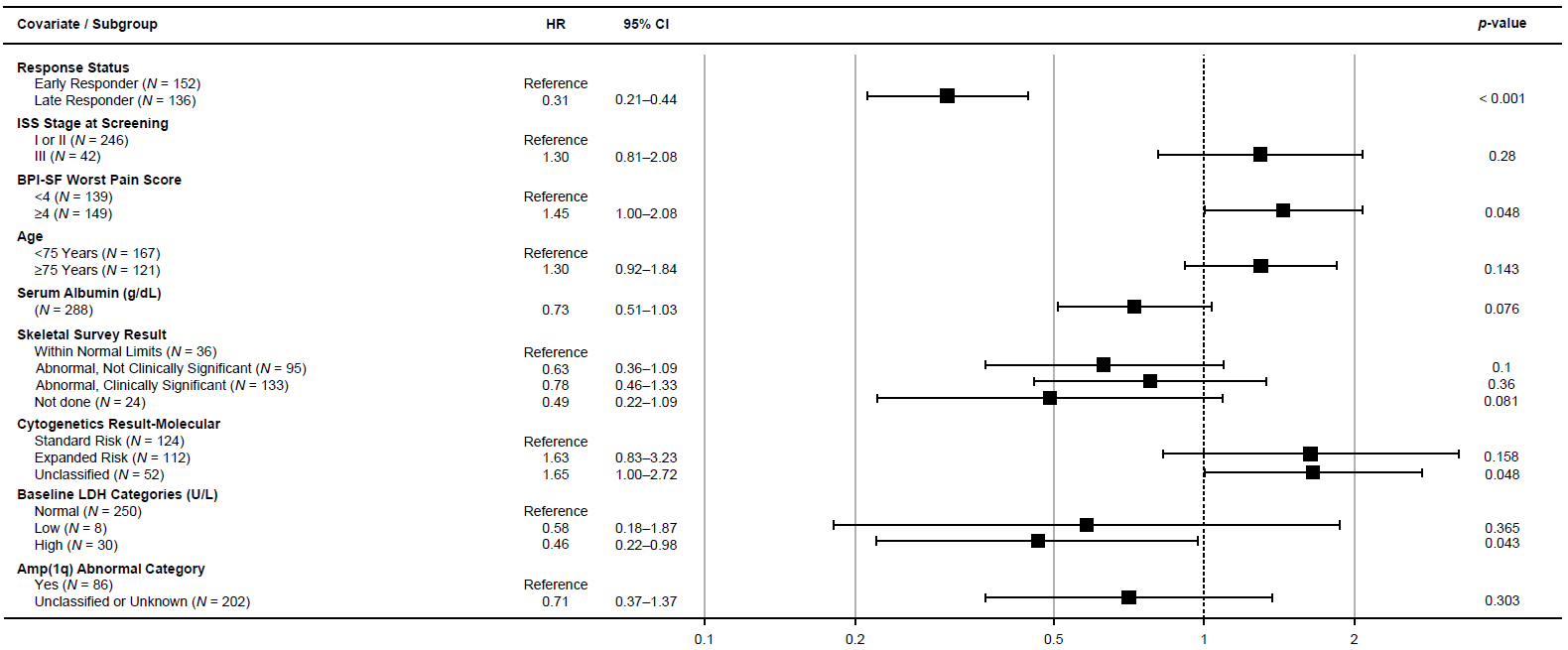


Cox proportional hazards model were used for HR values comparing baseline covariates of interest between late and early responders.

BPI-SF, Brief Pain Inventory-Short Form; CI, confidence interval; HR, hazard ratio; ISS, International Staging System; LDH, lactate dehydrogenase; PFS, progression-free survival; PR, partial response.

**REFERENCES**

1. Facon T, Venner CP, Bahlis NJ, Offner F, White DJ, Karlin L, et al. Oral ixazomib, lenalidomide, and dexamethasone for transplant-ineligible patients with newly diagnosed multiple myeloma. Blood. 2021;137(26):3616-28.
